# Fluctuations of Steady-State Accommodation Is a Marker for Screening Spasm of Near Reflex

**DOI:** 10.1167/tvst.10.11.9

**Published:** 2021-09-07

**Authors:** Shrikant R. Bharadwaj, Chandrika Ravisankar, Saujanwita Roy, PremNandhini Satgunam

**Affiliations:** 1 Brien Holden Institute of Optometry and Vision Sciences, L V Prasad Eye Institute, Hyderabad, India; 2 Prof Brien Holden Eye Research Centre, L V Prasad Eye Institute, Hyderabad, India

**Keywords:** accommodative spasm, photorefraction, pseudomyopia, RMS deviation, tonic accommodation

## Abstract

**Purpose:**

To determine the utility of root mean squared (RMS) deviations of steady-state accommodation as a noncycloplegic marker for spasm of near reflex (SNR) vis-à-vis regular refractive errors.

**Methods:**

Binocular steady-state responses of accommodation, pupil, and vergence of 20 patients with accommodative spasm subtype of SNR (SNR-A; 9–23 years) and 91 with regular refractive errors (29 emmetropes, 41 myopes, 21 hyperopes; 19–38 years) was recorded in the uncorrected refractive error state for 120 seconds using a dynamic (50 frames per second), infrared photorefractor. Mean and RMS deviation of raw data was calculated for three 20-second-long epochs and their diagnostic utility was determined using standard ROC curves.

**Results:**

RMS deviations of accommodation increased with mean refractive error in SNR-A (y = −0.23x + 0.38; *r^2^* = 0.69; *P* < 0.001) and regular refractive error (y = −0.02x + 0.10; *r^2^* = 0.14; *P* = 0.002) cohorts, albeit with steeper slope and higher y-intercept in the former rather than the latter cohort. RMS deviation of 0.19D reliably distinguished SNR-A from regular refractive errors with a sensitivity and specificity of 95.2% and 92.2%, respectively [mean (±1 SEM) area under ROC curve: 0.98 ± 0.01]. The sensitivity, specificity, and area under ROC curve for RMS deviations of pupil (66.7%, 80%, and 0.70 ± 0.09) and vergence (52.4%, 84.6%, and 0.68 ± 0.08) were smaller than accommodation.

**Conclusions:**

RMS deviations of steady-state accommodation is a robust noncycloplegic marker for differentiating SNR-A from regular refractive errors. Pupil and vergence fluctuations have limited utility in this regard.

**Translational Relevance:**

RMS deviations of accommodation may be easily obtained using commercial photorefractors, and the cut-off values reported herein may be implemented to identify SNR-A during refractive error screening.

## Introduction

Spasm of near reflex (SNR) is an umbrella term describing dysfunction of the near triad (accommodation, vergence, and pupils) with signs of manifest myopia that are largely eliminated with cycloplegia (pseudomyopia), fluctuations in visual acuity, vacillating retinoscopy reflex, and accommodative lead in dynamic retinoscopy as well as symptoms of blurred vision and asthenopia.^1^^,^^2^ Esodeviation of the eyes and pupillary miosis may also be seen in cases with prominent involvement of the vergence and pupil components of the near triad.^1^^,^^2^ The most commonly described subtype of SNR is accommodative spasm (referred herein as SNR-A), followed by the involvement of all three near triad components and isolated convergence spasm.^2^^,^^3^ Isolated pupil spasm is seldom reported. SNR-A is of primary concern to this study.

SNR is typically associated with the emotional state of the individuals,^1^^,^^2^ with the amount of time spent doing near work in high-stress environments,^2^^,^^4^ and following concussion.^5^ However, knowledge on the prevalence and incidence of SNR in the general population is very limited (see Discussion section for details). Those providing this data are for patients visiting a clinical setting, and they report a prevalence of SNR to be ∼2% to 6% among those with general binocular vision dysfunction.^1^^–^^3^^,^^6^ Population-level screening of SNR and differentiating it from regular refractive errors is important for two reasons. First, if refractive corrections are to be prescribed based on noncycloplegic refraction in a screening setup, then SNR could be misdiagnosed as high myopia and the wrong refractive correction may be prescribed.^7^^–^^12^ Second, if the referral criterion in a screening protocol is based on a certain visual acuity cutoff ^13^^–^^16^, some patients with SNR may pass this criterion owing to their fluctuations in visual acuity and may be missed from being appropriately diagnosed and managed.^1^^–^^3^ Presently, a limiting factor for the screening of SNR is that its diagnosis is critically dependent on the reduction in the eye's myopic refractive state before and after cycloplegia.^1^^–^^3^^,^^6^ Performing cycloplegic refraction is risky and impractical in screening settings.^1^^–^^3^^,^^6^ An alternate paradigm with high sensitivity/specificity, and that can be easily implemented in screening settings is therefore necessary for the early detection and management of SNR.

Recently, Bharadwaj et al. reported that the precycloplegic fluctuations of steady-state refraction in cases with SNR-A were at least four to five times larger in magnitude, relative to age-matched emmetropic controls.^17^ As the involvement was primarily related to accommodation, the corresponding fluctuations of pupils and vergence were unremarkable, relative to controls.^17^ This study demonstrated that the magnitude of accommodative fluctuations is a good noncycloplegic marker for screening SNR-A with high sensitivity in identifying this dysfunction and with high specificity in ruling out regular refractive errors (emmetropia, myopia, and hyperopia). Bharadwaj et al., in fact, demonstrated similar trends in SNR-A against a pilot data of five myopic and five hyperopic eyes, but the sample size was too small to perform any diagnostic accuracy analyses.^17^ The present study overcomes this lacuna by providing quantitative estimates of sensitivity and specificity in a larger cohort of patients with SNR-A and controls with wide range of manifest refractive errors.

## Methods

The study protocol adhered to the tenets of the Declaration of Helsinki and was approved by the Institutional Review Board of L V Prasad Eye Institute, Hyderabad. All subjects participated in the study after signing a written informed consent form. For participants <18 years old, assent was obtained while the consent form was signed by a parent or local guardian. The study cohort comprised of 20 cases (9–23 years old) with clinical diagnosis of SNR-A who visited the binocular vision and orthoptics clinic of the institute between 2016 and 2021 (Table); 91 controls with emmetropic (*n* = 29; 19–32 years old), myopic (*n* = 41; 20–38 years old), or hyperopic (*n* = 21; 20–30 years old) spherical equivalent refraction. Emmetropia was defined here as the spherical equivalent of refraction between ±0.5D of zero refractive error. The diagnosis and management of SNR of these patients are reported in detail in Roy et al. ^3^ and Bharadwaj et al.^17^ Briefly, diagnosis of SNR-A was confirmed by a >2.00D hyperopic shift in cycloplegic retinoscopy compared to noncycloplegic retinoscopy, with or without the disappearance of esodeviation with cycloplegia.^3^^,^^17^ Additional signs of vacillating retinoscopy reflex, reduction/fluctuations in visual acuity that were not proportionate to the refractive error, were also considered.^3^^,^^17^ SNR-A was managed through a combination of the modified optical fogging technique and pharmacological cycloplegia with weak and strong cycloplegics (depending on disease severity) along with vision therapy, all primarily aimed at relieving the spasm.^1^^,^^3^^,^^18^ All controls underwent a comprehensive eye examination and their refractive errors, if any, were corrected with spectacles or contact lenses. These controls had high-contrast distance visual acuity better than or equal to 20/20, stereoacuity better than or equal to 40 arc sec, and binocular vision parameters within the age-matched norms described in the literature.^19^ Subjects with associated ocular or systemic pathologies were excluded from the study. Presenting spherical equivalent refraction (SER) was obtained as an average of three readings from a closed-field autorefractor (Unique-RK, URK-800F, Daejeon, Republic of Korea) or from an approximate correction that resulted in a reversal of retinoscopy reflex.^3^ Presenting precycloplegic visual acuity of all participants was obtained using an electronic projection chart (Complog, Ver. 1.3.25.0, London, UK).^20^

**Table.** Details of Patients With SNR-A That Participated in This Study

Pt Num	Age (Yrs) | Gender	Clinical Diagnosis	Presenting SER (D)	Postcyclo SER (D)	Presenting VA	Postcyclo VA
P1	14 | M	Mild SNR	−7.90	0.5	0.3	0.0
P2	19 | M	Mild SNR	−1.80	0.6	1.6	0.0
P3	15 | M	Mild SNR	−5.50	0.3	1.1	0.1
P4	22 | M	Mild SNR	−3.00	0.5	0.0	0.0
P5	14 | F	Moderate SNR	−5.00	1.9	0.7	0.1
P6	15 | M	Mild SNR	−1.30	1.0	0.2	NR
P7	11 | M	Mild SNR	−3.00	0.0	0.1	0.0
P8	10 | F	Mild SNR	−5.00	1.25	1.0	NR
P9	9 | M	Mild SNR	−8.50	0.0	1.1	0.4
P10	13 | F	Mild SNR	−4.00	0.0	0.4	0.1
P11	12 | M	Mild SNR	−2.00	0.25	0.9	0.7
P12	13 | F	Mild SNR	−6.00	1.5	0.4	NR
P13	12 | M	Moderate SNR	−4.00	0.0	1.4	NR
P14	12 | M	Moderate SNR	−5.50	1.0	0.8	0.1
P15	23 | M	Moderate SNR	−3.00	2.0	0.4	0.0
P16	18 | M	Mild SNR	−3.00	1.25	0.1	0.0
P17	14 | M	Mild SNR	−5.00	0.25	1.5	0.0
P18	21 | M	Severe SNR	−2.00	1.50	0.5	0.1
P19	15 | F	Mild SNR	−5.00	−1.0	1.1	0.0
P20	14| M	Mild SNR	−4.00	1.0	0.6	0.0

The spherical equivalent objective refraction (SER) is shown for both the presenting and postcycloplegic conditions. High-contrast logMAR visual acuity (VA) is from the presenting condition of the patient. NR indicates that no data was recorded.

The data collection and analyses protocols are described in detail in Bharadwaj et al.^17^ Briefly, subjects binocularly fixated on a broadband spatial frequency line target at 2 m viewing distance (angular subtense: 2.8° height × 0.5° width at the nodal point of the eye) without their refractive correction in an otherwise dimly lit room. Given the broadband nature of the target, it was still visible, albeit blurred, to subjects with high refractive errors without their distance correction. These subjects were also instructed to fixate on the center of the target and were reminded to do so periodically throughout the experiment. Almost all the subjects that participated in the study were spectacle wearers. Obtaining photorefraction through spectacles can lead to erroneous results either due to reflections from the spectacles or due to magnification issues related to the spectacle correction.^21^ Correcting their myopia with soft contact lenses was also not appropriate, for, if this were not their habitual correction, it could result in short-term alterations in their accommodative and vergence behavior that may unduly influence the present study results.^22^

Subject's steady-state accommodation (i.e., refractive power in diopters relative to the photorefractor distance), binocular vergence eye position (in prism diopters), and pupil diameter (in millimeters) were recorded at 50 frames per second for 120 seconds using the PowerRef 3 eccentric, infrared photorefractor (Plusoptix GmBH, Nuremberg, Germany) from 1 m distance. Blinks in the raw data were either recorded as an empty cell for that frame or indicated by a value of –100 by the photorefractor. These were filtered out from the raw data before further analysis. Subsequent to this, the data of each subject was smoothed with a 100-ms-long running-average filter using custom written software in MATLAB (R2016a; The MathWorks Inc, Natick, MA). From the total 120 seconds worth of data collected, 60 seconds of the most continuous data with minimal blink artifacts was visually chosen by the investigator for further analysis. This segment was then spliced into three 20 second long epochs to calculate the mean and root mean squared (RMS) deviation. Refractive power estimates were scaled using an Indian ethnicity-specific scaling factor derived in Sravani et al. for increased accuracy and precision of outcomes.^23^ Binocular vergence was calculated as the difference between the two eyes’ gaze position obtained by tracking the first Purkinje image position with respect to the pupil center of each eye.^24^^,^^25^ A population-average Hirschberg ratio of 11.8°/mm was used as the conversion factor gaze position.^25^ These were converted from prism diopter units into meter angles (MA) by dividing the former with the subject's interpupillary distance, for ease of comparison with accommodation measurements.^24^^,^^25^ Pupil diameter was calculated by detecting pupil edges using built-in image processing algorithms.^26^ Overall, the experimental protocol did not take beyond 5 to 10 minutes to complete.

ROC curve analysis for the RMS deviations of each component of the near triad as the screening marker for SNR vis-à-vis regular refractive errors, was performed using IBM SPSS Statistics 20.0 (SPSS, Chicago, IL). Even though data were collected from both eyes, only the right eye was considered for analyses.^27^ Shapiro-Wilk test indicated nonnormal distribution of the outcome measures and hence nonparametric statistics were used for statistical analyses.

## Results

Table shows demographic details of patients with SNR-A that participated in this study. All but one patient had mild to moderate SNR-A while the remaining one had severe SNR, all based on the categorization described in Roy et al.^3^ The manifest refraction of these patients ranged from −1.25D to −8.0D of myopia and high-contrast logMAR visual acuities ranging from 0 to 1.6 logMAR (Table). All patients were relieved of SNR-A after cycloplegia or with the modified optical fogging^18^ either on the first postcyclopentolate visit (mild SNR) or on the first postatropine visit (moderate SNR).^3^ The one patient with severe SNR was continued on atropine eye drops and called for follow-up in three months’ time.^3^

**Figure 1.**
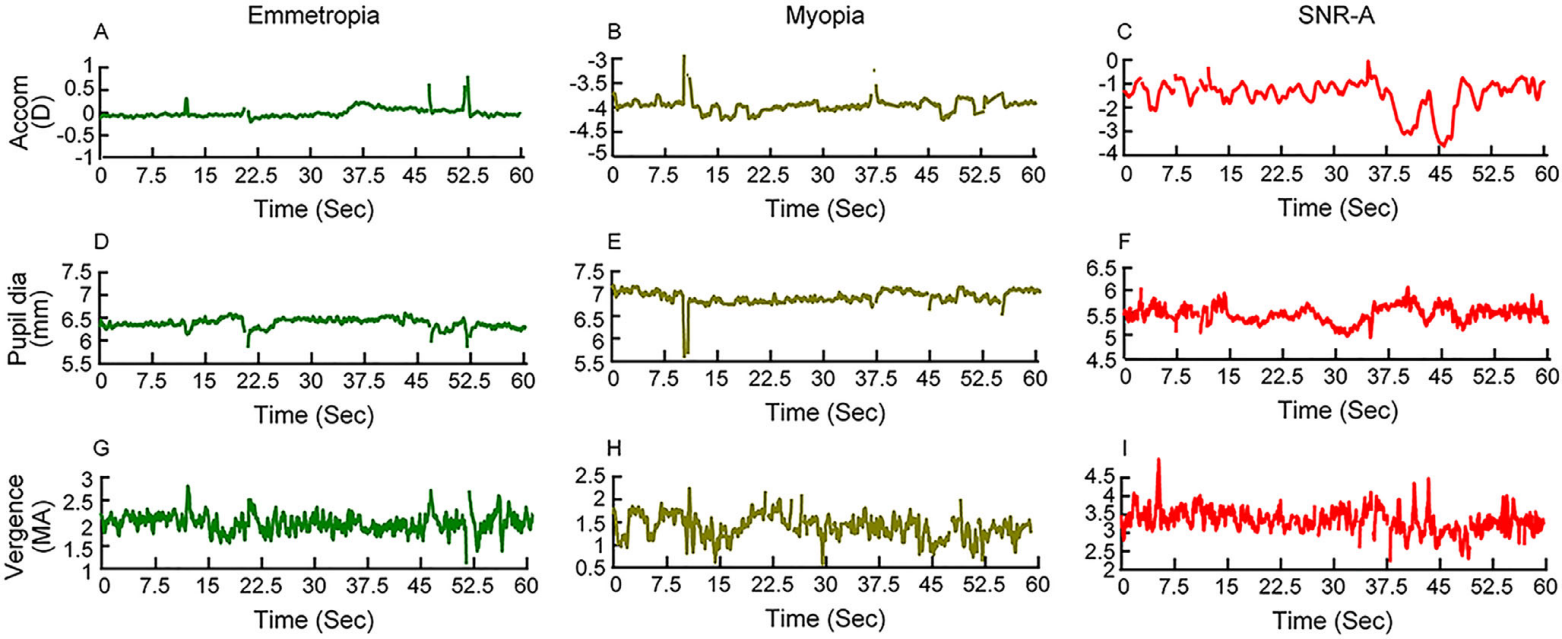
Raw traces of right eye's accommodation (panels A–C), pupil diameter (panels D–F), and binocular vergence eye position (panels G–I) plotted as a function of time for three representative subjects, one each with emmetropic refraction (*left panels*), myopic refraction (*middle panels*) and SNR-A (*right panels*). While data was collected for 120 seconds from each subject, data worth only 60 seconds is displayed here. The ordinate scales are different for each panel to best represent the details in the data. All data reported here were collected without the subject's refractive correction in an otherwise dimly lit room.

Figure 1 shows raw traces of accommodation (panels A–C), pupil diameter (panels D–F), and binocular vergence eye position (panels G–I) plotted as a function of time for three representative subjects, one each with emmetropic refraction, myopic refraction, and SNR-A. The raw traces showed larger fluctuations in the steady-state response of all three components of the near triad in the patient with SNR-A, relative to the two subjects with regular refractive errors (Fig. 1). This increase was most prominent for accommodation, followed by binocular vergence, and then pupil diameter (Fig. 1). Detailed Fourier-transformed temporal amplitude spectra of these fluctuations in controls and cases with SNR-A can be found in Bharadwaj et al.^17^

**Figure 2.**
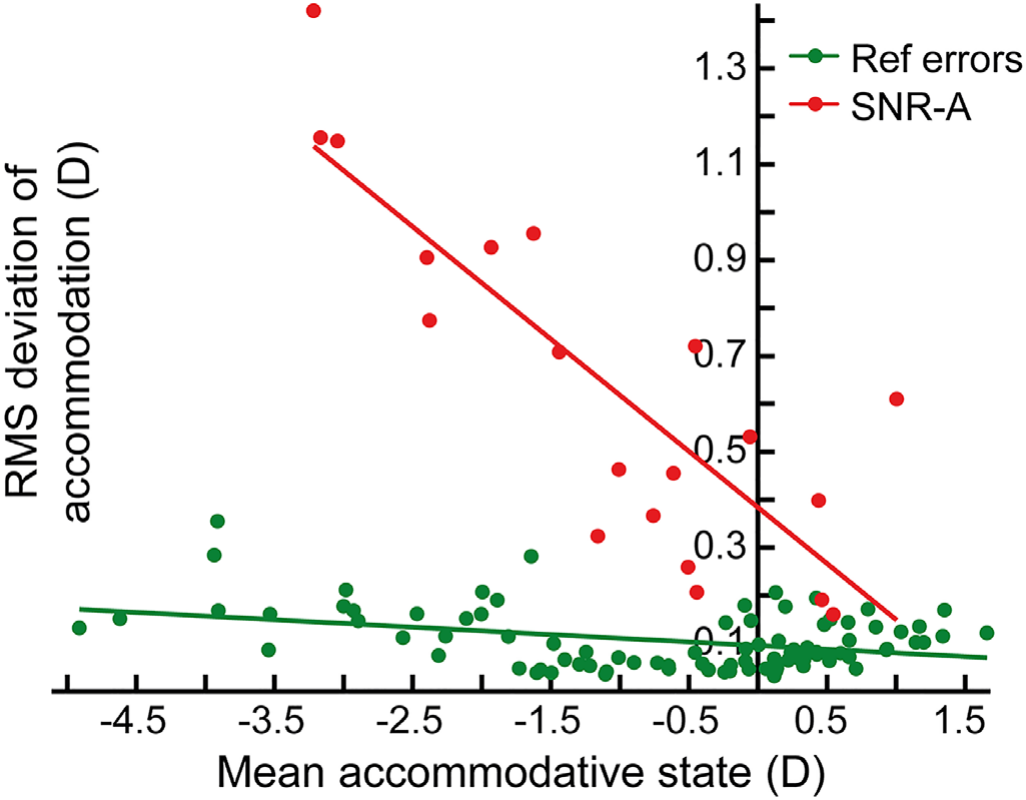
Scatter diagram of the RMS deviation of right eye's accommodation plotted as a function of the corresponding mean accommodative state in the SNR-A and regular refractive error cohorts.

The mean RMS deviation of steady-state accommodation of patients with SNR and regular refractive errors are plotted as a function of their mean accommodative state obtained from the photorefractor in Figure 2. Overall, the RMS deviations were significantly higher for the SNR-A cohort than for controls (two tailed Mann-Whitney *U* test, *P* < 0.05) (Fig. 2). The RMS deviations of accommodation of cases increased at the rate of −0.23D per unit increase in the mean accommodative state and with a y-intercept of 0.38D (*r^2^* = 0.69; *P* < 0.001) (Fig. 2). The RMS deviations of accommodation of those with regular refractive errors also increased significantly with their mean accommodative state, albeit with a shallower slope and smaller y-intercept than that of SNR-A; explaining only 14% of the variance in the data (y = −0.02x + 0.10; *r^2^* = 0.14; *P* = 0.002). The mean (±1 SEM) pupil diameter of cases with SNR-A (5.6 ± 0.9 mm), emmetropes (6.0 ± 0.8 mm), myopes (5.8 ± 0.9 mm), and hyperopes (5.7 ± 0.8 mm) were not significantly different from each other (one-way ANOVA, *P* = 0.33). The mean (±1 SEM) vergence state of the eye was however significantly different across cohorts (one-way ANOVA, *P* < 0.05), with post hoc Bonferroni analysis indicating that the data of the hyperopic cohort (2.52 ± 0.74 MA) to be significantly different from the myopic cohort (1.53 ± 0.90 MA) (*P* < 0.001) and the SNR-A cohort (1.72 ± 0.95 MA), and the data of the emmetropic cohort (2.15 ± 0.70 MA) to be significantly different from the myopic cohort (*P* = 0.02). None of the other pairwise comparisons were statistically significant.

**Figure 3.**
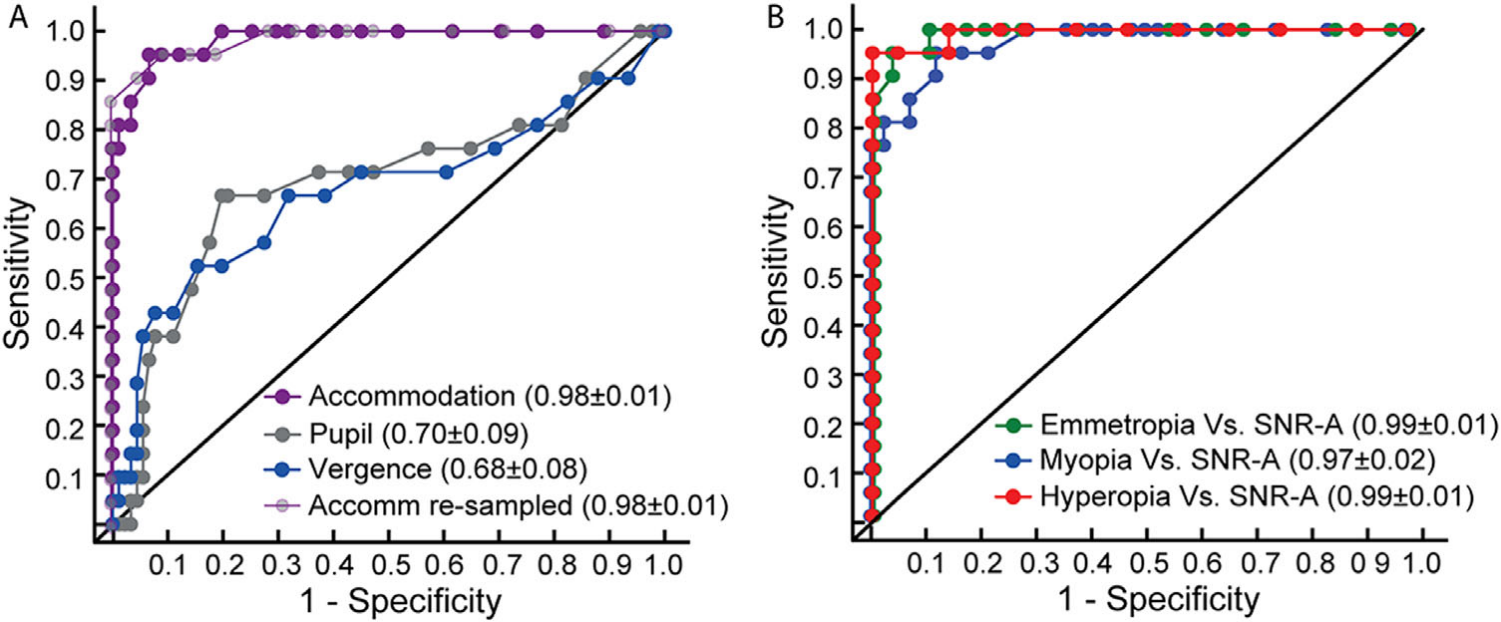
Panel A shows receiver operating characteristics (ROC) curves for RMS deviation of accommodation, pupils, and vergence along with the respective mean (±1 SEM) area under the curve values. The ROC curve for accommodation with the resampled data of the regular refractive error cohort is also showed in this panel. Panel B shows the ROC curves for three different refractive error cohorts compared against the SNR cohort. All other details are same as panel A.

The ROC curve of the RMS deviation of accommodation as a marker for discriminating cases of SNR-A from those with regular refractive errors is shown in Figure 3. The mean (±1 SEM) area under this ROC curve was 0.98 ± 0.01 and the Youden's J index was highest (0.89) for an RMS deviation value of 0.19D (Fig. 3A). The sensitivity and specificity of the RMS deviation of accommodation in discriminating cases from controls for this value of Youden's J index was 95.2% and 93.4%, respectively. The steady-state fluctuations of pupil and vergence did not show as good a discriminatory ROC curve as accommodative fluctuations between cohorts (Fig. 3A). The mean (±1 SEM) area under ROC curve for pupil fluctuations was 0.70 ± 0.09 and the highest value of Youden's J index (0.47) was obtained for a pupil fluctuation of 0.19 mm (Fig. 3A). The sensitivity and specificity at this value of Youden's J index were only 66.7% and 80.2%, respectively. Similarly, the mean (±1 SEM) area under ROC curve for vergence fluctuations was 0.68 ± 0.08, and the highest value of Youden's J index (0.37) was obtained for a vergence fluctuation of 0.30 MA (Fig. 3A). The sensitivity and specificity at this Youden's J index were 52.4% and 84.6%, respectively.

The number of subjects in the regular refraction error and SNR-A cohorts were widely different from each other in this study (*n* = 91 and 20 for the former and latter, respectively). This was so because of the limited number of patients with SNR-A that visited the study site during the indicated study duration and the larger number of samples in the regular refractive error cohort required to match the corresponding range observed in SNR-A. To determine if the unequal sample size had an impact on the ROC analysis reported earlier, the regular refractive error cohort was resampled to 20 subjects to match the number of participants in the SNR-A cohort and the diagnostic indices were redetermined from the revised ROC analysis. Resampling of the regular refractive error cohort was achieved by sorting the data in ascending order of their mean refraction, as recorded by the photorefractor, and including data of every fourth subject for this analysis until the sample size of 20 was reached. This resampling technique resulted in a range of refractive errors (+1.04D to −4.91D) that matched the refractive error range of the SNR-A cohort (+1.00D to −4.99D). The ROC analysis on this resampled data resulted in results that were nearly identical to the original analysis. The mean (±1 SEM) area under ROC curve for accommodative fluctuations was 0.98 ± 0.01, and the highest value of Youden's J index (0.86) was obtained for a pupil fluctuation of 0.19D (Fig. 3A). The sensitivity and specificity at this value of Youden's J index were 95.2% and 90.5%, respectively. This suggests that the uneven sample size of the two cohorts had only a limited impact on the ROC analysis aimed at testing the utility of RMS fluctuations of accommodation as a marker for differentiating SNR-A from regular refractive errors.

An additional analysis was performed to determine if the ability of accommodative fluctuations to differentiate SNR-A from regular refractive errors continued to exist even when the data of controls was divided into separate cohorts of emmetropes (*n* = 29), myopes (*n* = 41), and hyperopes (*n* = 21). Figure 3B plots the corresponding ROC curves for this analysis, and the results were expectedly not different from the data shown in Figure 3A. The mean (±1 SEM) area under ROC curve for the emmetropic versus SNR-A comparison was 0.99 ± 0.01, for the myopic versus SNR-A comparison was 0.97 ± 0.02, and for the hyperopic versus SNR-A comparison was 0.99 ± 0.01. The highest value of Youden's J index ranged from 0.83 to 0.95D for all three comparisons, with the myopic cohort showing the lowest Youden index and the hyperopic cohort showing the highest Youden index. The sensitivity and specificity at these values of Youden's J index was 95.2% and ranged from 87.8% to 100% across the three cohorts, respectively (Fig. 3B).

## Discussion

### Fluctuations of Refraction in SNR

The present results indicated that temporal variations in the refractive state of the eye may be used as a noncycloplegic marker for differentiating SNR-A from true refractive errors with high sensitivity and specificity (Fig. 3). The condition, once detected using this marker in screening settings, may be confirmed in specialty clinics using gold-standard cycloplegic refraction and subsequently managed using present clinical protocols.^2^^,^^3^ These results are not surprising, for fluctuations in the refractive state of the eye are routinely observed while examining patients with SNR, and they are used by experienced clinicians as a qualitative sign to confirm their diagnosis. The most direct clinical manifestation of these refractive fluctuations is the vacillating retinoscopy reflex in SNR that renders the endpoint of objective refraction very challenging and/or large variations in the autorefractor output upon repeated measurements in SNR.^2^^,^^3^ Patients also subjectively report a temporal fluctuation in their vision as a part of their presenting history and/or during their clinical visual acuity assessment, indirectly reflecting the underlying fluctuations in the eye's optical power.^2^^,^^3^ The present results also demonstrated that steady-state fluctuations of the pupil and vergence eye position are not as much of diagnostic value for differentiating SNR from those with regular refractive errors (Fig. 3). This is not surprising considering that the SNR cohort recruited for this study were of the accommodative spasm subtype and the raw data of these responses showed limited differences between the two cohorts (Fig. 1). Perhaps, repeating this study in a cohort of patients with convergence-spasm subtype of SNR may yield better ROC curves for these two parameters of the near triad.^28^^,^^29^ On a related note, all the measurements obtained in this study and in the previous one by Bharadwaj et al.^17^ were under binocular viewing conditions, where both blur-driven accommodation and disparity-driven vergence were active. The independent role of these two elements in determining the characteristics of SNR, especially the fluctuations in refraction, cannot therefore be ascertained from the present study. Future studies may compare the characteristics of SNR-A under monocular and binocular viewing conditions to gain further insights into this issue.

The marginal increase in the magnitude of refractive fluctuations in myopes vis-à-vis emmetropes in Figure 2 of this study is similar to the observations of several previous studies.^30^^–^^33^ In fact, the rate of increase in the RMS deviations of accommodation with the subject's spherical equivalent refraction is quantitatively similar between the present study and Harb et al.^31^ Both studies documented ∼0.02D increase in fluctuations per diopter increase in myopic refraction for 0.5D and 1.5D accommodative demands, respectively. The rate of increase in the magnitude of these fluctuations with the underlying refractive error was however very shallow compared to those with SNR (0.23D increase in fluctuations per diopter increase in manifest myopic refraction) and explained only a minority of the variance in the data (14%) relative to those with SNR (69%) (Fig. 2), implying that even while the manifest refractive error may be similar in the two cohorts, the fluctuations in this manifest refraction will be orders of magnitude higher in the latter than in the former cohort (Fig. 2). This small increase in the RMS deviations of accommodation with increasing myopia also explains why the ROC parameters for the myopic versus SNR-A comparison were marginally smaller than that of the emmetropic and hyperopic comparisons (Fig. 3B). The quantum of difference in the RMS deviations of accommodation would have reduced with increasing myopia, thus affecting the specificity of this parameter in distinguishing regular myopes from those with SNR-A. However, this loss was only marginal and is of little practical consequence to the proposed use of accommodative fluctuations as a noncycloplegic marker for SNR-A. The pattern of results also suggest different sources/physiological reasons for these fluctuations in the myopic and pseudomyopic cohorts—refractive fluctuations in myopia may be attributed to the larger depth of focus arising from poor sensitivity to blur,^30^^–^^33^ while the refractive fluctuations in pseudomyopia may arise from abnormal gain and decay time constants of tonic accommodation.^17^ Fluctuations of steady-state accommodation have also been found to be somewhat higher in children with uncorrected hyperopia, relative to emmetropes, and this has been attributed to immature blur detection capabilities and immature motor accommodation of this cohort.^34^ The present study did not observe any such patterns, although the range of hyperopia included in this cohort was relatively narrow (<2D), and the participants were all adults, presumably with mature blur detection and motor accommodative capabilities.

### Photorefraction as a Tool for Screening SNR

Any healthcare screening device should have high sensitivity/specificity, low cost, portability, be noninvasive, allow rapid implementation, and require limited dependency on a technically trained human resource. Commercially available photorefractors and photoscreeners (e.g., Spot Vision Screener, Welch Allyn, Chicago, IL, USA and plusoptix, S16, Plusoptix GmbH, Nuremberg, Germany) meet all the criteria for screening refractive anomalies of the eye.^35^^,^^36^ They are noninvasive with a measuring distance of 1 m, handheld, able to obtain data from both eyes synchronously rapidly (20 ms for one frame in dynamic photorefractors and no more than a few seconds in photoscreeners) and can be operated by nontechnical staff with minimal training. Additionally, these devices are quite tolerant to head movement and, thus, do not require precise head stabilization. They also provide valuable data on all three components of the near triad simultaneously, potentially screening for strabismus.^37^ The results of the present study indicate that the scope of these devices may be expanded from screening of regular refractive errors to include other refractive anomalies like SNR using the RMS deviation of steady-state accommodation as its marker (Fig. 3). Minor modifications to the detection/analysis algorithm of these commercial devices may, however, need to be made before they become ready for screening of SNR. The present photoscreeners provide only a mean estimate of the refractive error, without any measure of their fluctuation over time. The proposed RMS deviation of accommodation is inherently a time-varying function, and it will require these devices to capture and analyze time-series data. The research version of some of these photorefractors, such as the Plusoptix PowerRef 3 used in this study, already has this capability built into their algorithm.

Refractive error estimates of the eye obtained using photorefraction is critically dependent on the absolute and relative calibration of the luminance profile formed across the pupil into units of diopters.^23^^,^^38^^–^^41^ The former calibration ensures accurate estimates of the absolute refractive state of the eye (e.g., a 3D myopic eye is recorded as such with the photorefractor) while the latter ensures accurate estimates of change in the refractive state of the eye over time (e.g., refractive power change during accommodation).^39^ Obtaining an absolute calibration on each eye is not trivial, let alone implementing it in a screening setting. Relative calibration factors that are ethnicity-specific have already been published in the literature, and they could be more readily applied to the raw data obtained using commercial photorefractors, as was done in the present study.^23^ The proposed screening marker for SNR—the RMS deviations of accommodation—will be accurate and error free once the data are scaled using the ethnicity-specific relative calibration factor. The data presented in this study were scaled only by the relative calibration factor of photorefraction and, therefore, the absolute values of refraction reported in this study should be interpreted with caution (Figs. 1 and 2). This issue, combined with the fact that the spherical equivalent refractions reported in SNR-A indicated only an approximate estimate of the subject's refractive error, may explain the mismatch in the values of refraction reported in Table and Figure 2 of this study.

### Fluctuations of Refraction from Built-in Measurement Errors of Photorefraction

The results of the increase in the RMS deviations of accommodation reported in Figure 3 of this study is unlikely to arise from three potential sources of errors built into this experiment. First, the variability of refractive power estimated using photorefraction may increase with the underlying refractive error owing to increase in multiplicative noise with the brightness of reflex across the pupil. However, this variability increase tends to be prominent only for higher refractive errors as observed in the sample defocus calibration curve shown in Sravani et al.^23^ Further, any increase in the measurement variability of photorefraction will not be able to explain the differential RMS deviations of accommodation found between the myopic and SNR-A cohorts that had similar range of manifest refractive error (Fig. 3). Second, variability in photorefraction also tends to increase with pupil miosis because of sparse distribution of pixel values inside the pupil to calculate the luminance profile slope. The range of pupil diameters in this study, however, were not significantly different between the control and SNR-A cohorts (3.72–6.80 mm) and, thus, whatever increase in the variability of refraction estimates using photorefraction would be expected to be equal between the two cohorts. Taken together, it appears highly unlikely that the reported increase in the RMS deviations of accommodation may be related to the underlying measurement variability of photorefraction. Third, all measurements were obtained in this study with the subject's refractive error uncorrected, for the reasons explained in the methods section. The fixation target would have therefore appeared blurred to subjects with high refractive error and this may have increased their fixation instability vis-à-vis their emmetropic or low refractive error counterparts. This increased fixation instability may have in turn contributed to the increased variability of refraction reported in this study by potentially tracking peripheral refraction. This possibility appears unlikely as well, for the RMS deviations of eye position did not show any significant trend as a function of the subject's refractive error in the present study (linear regression slope: −0.002 MA/D), indicating that subjects with larger uncorrected refractive error did not necessarily have larger fixation instability and, therefore, larger RMS deviations of accommodation. This observation is also in line with Ukwade and Bedell who showed only a marginal increase in the fixation instability (from 8 to 12 arc min) from the no blur to 4D blur condition.^42^ This fixation instability is well within the range over which refractive error estimates have been shown to be unaltered in photorefraction.^24^

### Pseudomyopia Versus SNR-A

Several large-scale epidemiological studies in the past two decades have provided prevalence estimates of pseudomyopia in children.^7^^–^^12^^,^^43^^,^^44^ Pseudomyopia was defined in these studies as the elimination of myopic refraction or a hyperopic refraction following cycloplegia, relative to their precycloplegic manifest myopic refraction.^7^^–^^12^^,^^43^^,^^44^ The average pseudomyopia reported in these studies ranged from 0.3 to 0.84D in teenage children and slightly larger in younger children,^7^^–^^12^^,^^43^^,^^44^ with prevalence values ranging from 3% to 34%.^7^^–^^12^^,^^43^^,^^44^ We believe that the pseudomyopia reported in these studies are different from the entity of SNR described in the present study for the following reasons. First, elimination of manifest myopia with cycloplegic refraction is certainly a hallmark of SNR, and, in this sense, the manifest myopic refraction seen in the previous studies and in SNR are both pseudomyopia. However, pseudomyopia is only one of the many signs and symptoms of this dysfunction, as described in the introduction section.^1^^,^^2^ These associated signs and symptoms may be absent in the pseudomyopic children reported in the aforementioned studies. Second, as acknowledged in several of these studies, the shift in cycloplegic refraction toward hyperopia may also reflect the latent hyperopia that is relatively strong in these children.^10^^,^^12^ An allowance of up to 0.75D is usually provided in the final clinical acceptance of the patient following cycloplegic refraction for this reason.^45^^,^^46^ Myopic refraction in SNR-A is thought to arise from abnormally high gain and very slow decay time constant of the tonic accommodation controller,^17^ quite different from the latent hyperopia in the pseudomyopic children of these studies. Third, expectedly, the magnitude of pseudomyopia in the aforementioned studies declined with the patient's age,^7^^–^^12^^,^^43^^,^^44^ while the pseudomyopia in SNR may only show a weak age-related trend until the onset of presbyopia,^17^ as evident from the pre- and postcycloplegic refraction of patients in Table of this study. Taken together, we believe that the prevalence of SNR still remains unknown in the literature and that the RMS deviations of steady-state accommodation may be a robust noncycloplegic marker for screening this dysfunction and may be implemented effectively using photorefraction technology.

In conclusion, the RMS deviations of steady-state accommodation is a robust noncycloplegic marker for differentiating SNR-A from regular refractive errors. This marker may be relatively easily implemented in screening settings that use commercial photorefractor technology.
